# Chinese organic rice transition spatial econometrics empirical analysis

**DOI:** 10.1371/journal.pone.0297784

**Published:** 2024-04-11

**Authors:** Zhuo Luo, Yongxin Huang

**Affiliations:** 1 Guangzhou Xinhua University, Guangzhou, China; 2 Nanning Normal University, Nanning, Guangxi, China; National Technical University of Athens: Ethniko Metsobio Polytechneio, GREECE

## Abstract

Based on the integrated model of Super-SBM model, spatial Durbin model (SDM) and Grey neural network model, this paper analyzes the panel data of various provinces in China from multiple angles and dimensions. It was found that there were significant differences in eco-efficiency between organic rice production and conventional rice production. The response of organic rice to climate change, the spatial distribution of ecological and economic benefits and the impact on carbon emission were analyzed. The results showed that organic rice planting not only had higher economic benefits, but also showed a rising trend of ecological benefits and a positive feedback effect. This finding highlights the importance of organic rice farming in reducing carbon emissions. Organic rice farming effectively reduces greenhouse gas emissions, especially carbon dioxide and methane, by improving soil management and reducing the use of fertilizers and pesticides. This has important implications for mitigating climate change and promoting soil health and biodiversity. With the acceleration of urbanization, the increase of organic rice planting area shows the trend of organic rice gradually replacing traditional rice cultivation, further highlighting the potential of organic agriculture in emission reduction, environmental protection and sustainable agricultural production. To this end, it is recommended that the Government implement a diversified support strategy to encourage technological innovation, provide guidance and training, and raise public awareness and demand for organic products. At the same time, private sector participation is stimulated to support the development of organic rice cultivation through a public-private partnership model. Through these measures, further promote organic rice cultivation, achieve the dual goals of economic benefits and environmental benefits, and effectively promote the realization of double carbon emission reduction targets.

## 1 Introduction

Climate change is an increasingly serious problem and global greenhouse gas emissions continue to increase, leading to significant changes in the climate system with serious economic, social and environmental impacts [1]. Governments and businesses have taken a variety of measures to address climate change [2–4], which includes low-carbon development, is an important strategy that has received a lot of attention. China, as one of the world’s largest greenhouse gas emitters, is facing serious climate change challenges and has taken proactive measures to address the problem. The Chinese government has set peak carbon and carbon neutral targets to combat climate change [5], with plans to achieve peak carbon emissions by 2030 and carbon neutrality by 2060. In this context, the transformation of organic rice production in China, one of the world’s largest rice producers, has become a topic of great concern. The possibility and benefits of this transformation become the focus of research.

In recent years, studies on agricultural carbon emissions have become increasingly detailed, and many scholars have discussed their causes [6] and spatio-temporal evolution [7, 8], Influencing factors [9–11], emission reduction pathways [12] and intervention effects [13]. Agricultural carbon emissions have previously been reported to be associated with irrigation, farming, agricultural diesel, agricultural film, pesticides, and fertilizers [14]. The transition from high carbon to low carbon agriculture is essential for developing green agriculture and mitigating global climate change [15]. Studies have shown that compared with conventional agriculture, organic agriculture can use energy more efficiently, reduce greenhouse gas emissions, reduce nitrate and ammonia losses, and contain more soil organic matter, contributing to increased biodiversity [16–21]. In contrast, conventional agriculture is thought to consume more energy, produce more NO emissions, lead to more nitrate loss when measured globally, more ammonia emissions, and have higher eutrophication and acidification potential [18, 19].

In China, rice accounts for almost 30% of global production and 28% of global consumption [22]. The study found that more than 50% of the global population relies on rice [23] to meet their dietary needs, and its production plays an important role in shaping the environmental profile of agricultural systems [24]. In this context, the study of the ecological efficiency of organic rice and its impact on carbon emissions has important reference significance for in-depth understanding of the overall ecological benefits and environmental impacts of organic agriculture. The emerging empirical literature on the adoption of organic agriculture considers spatial data heterogeneity and dependency characteristics. Bartolini and Vergamini [25] found that the location of farms on the plains has a negative impact on the projected percentage of organic production in Tuscany, and Chen et al.[26] found a positive spatial dependence on environmentally friendly technologies such as organic agriculture in China. Longo et al.[27] showed that soil conditions play an important role in determining the geographical distribution of organic farms. Taken together, most of these studies suggest that the distribution of organic agriculture is spatially dependent. Despite the growing literature on the spatial relevance of agriculture, organic agriculture is a relatively new mode of agricultural production, and the number of studies on organic rice production is still small compared to traditional agriculture. And because the technology and practice of organic agriculture are different from that of traditional agriculture, farmers need time to adapt to the new methods and technologies, which leads to the spatial distribution and regional differences in organic rice production. Assessing the economic impact of organic rice production also requires considering more factors and conducting more sophisticated analyses. With the development and increasing interest in organic agriculture, our research has important implications for promoting sustainable agricultural development, optimizing resource use and agricultural economic growth. Based on the perspective of ecological benefit and spatial econometrics, this study used a combination model of Super-SBM model, spatial Durbin model and grey dynamic model to study the transformation of organic rice in China.

It is found that organic rice planting has high economic and environmental benefits, and the ecological benefits are increasing year by year. The urbanization process promotes the increase of organic rice planting.

The contribution of this work was as follows. (i) Using provincial panel data and a relaxation-based econometric model, this study provides a new analytical framework to accurately assess the ecological benefits of organic and conventional rice, providing important empirical support for organic agriculture research. (ii) Provide important evidence of the high economic and environmental benefits of organic rice cultivation, demonstrate the feasibility and advantages of organic agriculture, and provide important guidance and initiatives for achieving agricultural sustainable development and environmental protection goals. (iii) Broaden the understanding of the impacts of urbanization and the transformation of agricultural production practices, and provide guidance for agricultural policy makers to promote the development of organic rice cultivation and sustainable agricultural development. (iv) Based on its findings, the study also provides policy recommendations aimed at promoting sustainable agricultural development, promoting environmental protection and addressing climate change challenges, providing important strategic directions and policy frameworks for achieving the dual carbon goal and sustainable development of agriculture.

## 2 Literature review

There are relatively few existing studies that address the effects of organic rice eco-efficiency and organic rice eco-efficiency on carbon emissions. The existing studies [28–30] have mainly analysed the effects of organic farming eco-efficiency and organic farming eco-efficiency on carbon emissions. Mainstream studies have consistently demonstrated the potential advantages of organic farming systems in reducing carbon emissions compared to conventional farming systems. However, it should be noted that the carbon emission reduction effect of organic farming systems may be influenced by various factors, such as geography, climate, soil type, crop type, and management practices, so the complexity and uncertainty of various factors need to be accounted for, and more in-depth research and empirical analysis need to be conducted for specific application and promotion. Advances in research on the eco-efficiency of organic agriculture suggest that organic agriculture has the potential to protect the environment and improve ecosystem health and sustainable agricultural production [31].

Scholars have addressed the ecological effects of organic agriculture on carbon emissions, which is a hot research topic in the field of environmental science and agroecology. First, organic agriculture usually adopts natural ecological cycles, biodiversity conservation, soil health and other management measures to achieve carbon emission reduction [32–34]. Second, organic farming systems are usually more dependent on ecological cycles and ecological balance, reducing dependence on fossil energy sources and thus reducing greenhouse gas emissions, especially carbon dioxide [35, 36]. In addition, it has been found that organic farming systems typically focus more on maintaining ecosystem complexity and stability in farm and crop management, thereby increasing the potential for carbon emission reduction [37, 38]. The potential advantages of organic farming systems over conventional farming systems in reducing carbon emissions can be attributed to their ecologically sustainable management practices, their reliance on ecological cycles and balances, and the maintenance of ecosystem complexity and stability [39, 40]. Many scholars have explored the potential of organic farming in improving eco-efficiency by comparing the ecological effects of organic and conventional agriculture. Some literature indicates that organic agriculture can positively affect ecosystems and the environment by improving soil quality, promoting biodiversity, eliminating the use of chemical pesticides and fertilizers, and protecting water resources [41–45]. For example, studies have shown that organic agriculture can promote the accumulation of soil organic matter and improve soil fertility and water retention, thereby combating soil erosion and improving soil quality [46–48]. In addition, organic agriculture can reduce dependence on chemical pesticides by providing biodiversity habitats that promote the survival and reproduction of natural enemies and beneficial insects, thus enabling natural biological control [21]. However, some literature questions the eco-efficiency of organic farming. Some studies have argued that organic farming is relatively inefficient because of its smaller scale of production and lower level of technology and management, resulting in lower yields and higher costs [49]. In addition, organic farming may face challenges in resisting pests and diseases and protecting crops from climate change and other production risks, which may negatively affect its eco-efficiency [50, 51].

When scholars explore the issue of carbon peaking and carbon neutrality, the proposed paths to carbon neutrality mainly include low-carbon technology progress, industrial restructuring, regional technological balance, carbon emission rights market reform, and environmental regulation [52, 53]. Although the above paths may cause potential production losses in the short term, they can still achieve a win‒win outcome for both the environment and economic development in the long term. In the context of the gradual development of the pilot policy of carbon emissions trading, an increasing number of scholars have paid attention to this policy and emphasized the importance of carbon emissions trading for carbon neutrality [54]. From the unique perspective of economic agglomeration, scholars have found that it has a positive impact on carbon emissions, further broadening the path to achieving carbon neutrality. The literature has set different evaluation criteria for various potential carbon neutral pathways based on different research perspectives. In summary, the evaluation criteria for carbon neutral pathways are as follows: first, representative studies have evaluated the equity of carbon neutral pathways using methods such as the coefficient of variation, Gini coefficient and Thayer index [55, 56]; second, the economic consequences and environmental consequences of different carbon neutral pathways were evaluated from various aspects such as economy, industry, energy, environment, policy, and management [14]; third, the relationship between carbon emissions and economy was measured, and the carbon neutral pathways were evaluated by methods such as the Tapio decoupling model [57, 58]; fourth, the performance of different carbon neutral pathways is demonstrated by using comparative analysis [59]; fifth, the sustainability of carbon neutral pathways is analysed by considering the economic environment and other factors [60].

In research targeting the ecological benefits of organic rice and its impact on carbon emissions, the study of the carbon emission reduction effect is additionally influenced by various factors due to the complexity, dynamics and uncertainty of organic farming systems [61, 62]. Therefore, this study uses innovative ecosystem theory and social ecology theory to explore the ecological benefits of organic rice and its effect on carbon emissions from multiple perspectives based on theoretical characteristics.

Innovation ecosystem theory understands and explains the innovation process from an ecological perspective [63]. It emphasizes that innovation not only takes place in isolation within a firm but also occurs in a complex ecosystem that includes the interaction and influence among multiple participants, such as firms, universities, research institutions, governments, social organizations, and consumers [64, 65]. The core ideas of the theory include the following. 1. Innovation ecosystems are complex and dynamic: there are complex interrelationships and interactions among participants in an innovation ecosystem, and these relationships and interactions evolve over time and in response to changes in the environment. Therefore, the innovation ecosystem is a dynamic system that requires constant adaptation and adjustment. 2. The innovation ecosystem is sharing-oriented and cooperative: innovation is often achieved by sharing resources, knowledge, and information among participants in an innovation ecosystem and through cooperation and collaborative innovation. This cooperativeness is not limited to cooperation among enterprises; it also includes cooperation among various participants, such as universities, governments, and social organizations. 3. The innovation ecosystem has an ecological niche and ecological diversity: different participants in the innovation ecosystem play different roles in the ecological niche, forming ecological diversity. The ecological niche among different participants includes not only economic roles but also social, cultural, and political roles in many dimensions. This ecological diversity is crucial to the stability and sustainability of the innovation ecosystem. 4. The innovation ecosystem is self-adaptive and self-organizing: the innovation ecosystem has the ability of self-adaptation and self-organization and can self-adjust and self-optimize in the case of changes in the external environment to maintain the stability of the system and continuous innovation [63, 66].

Social ecology theory is a theory that understands and explains human social behaviour and social systems from an ecological perspective [67]. It emphasizes the interrelationships and interactions between social systems and the natural environment, viewing society and the environment as an interdependent ecosystem [68, 69]. The core ideas of the theory include the following. 1. Interdependence of social system and the natural environment: social ecology believes that social system and natural environment are interdependent, and social behaviour and social structure are constrained and influenced by the natural environment, while the natural environment is also influenced by social behaviour and social structure. There are complex interrelationships and interactions between social systems and the natural environment. 2. Social systems’ use and influence on environmental resources: social ecology is concerned with the use and influence of social systems on environmental resources, including energy, water resources, land use, ecosystem services, and other aspects. It studies how social behaviour affects the natural environment and how the needs of the social system are met through the use of environmental resources. 3. Impact of social structure and social behaviour on the environment: social ecology studies the impact of social structure and social behaviour on the environment, including aspects such as population, social organization, and social culture. The way social structure and social behaviour use and manage environmental resources, as well as the impact on environmental policy and planning, are important elements of social ecology’s concern. 4. Sustainable development and environmental justice: social ecology emphasizes the importance of sustainable development and environmental justice, including how to achieve a balanced, equitable, and sustainable relationship between social systems and the natural environment and how to achieve environmental justice through environmental policy and management [68, 69].

## 3 Theory and model

Organic rice and conventional rice are two types of rice that differ in the process of cultivation and production. Traditional rice refers to rice grown by conventional agricultural methods. In traditional rice farming, farmers often use chemically synthesized pesticides and fertilizers to control pests and diseases and increase yields. Genetic modification technology may also be used in traditional rice production, through transgenic rice varieties to improve disease resistance and resistance to pests. Organic rice refers to rice grown on the basis of organic agricultural principles. Organic agriculture is an agricultural system that focuses on ecological balance, environmental protection and sustainable development. Studies have shown that organic agriculture can promote the accumulation of soil organic matter, improve soil fertility and water retention, thereby combating soil erosion and improving soil quality [46–48].

**Fig 1:**
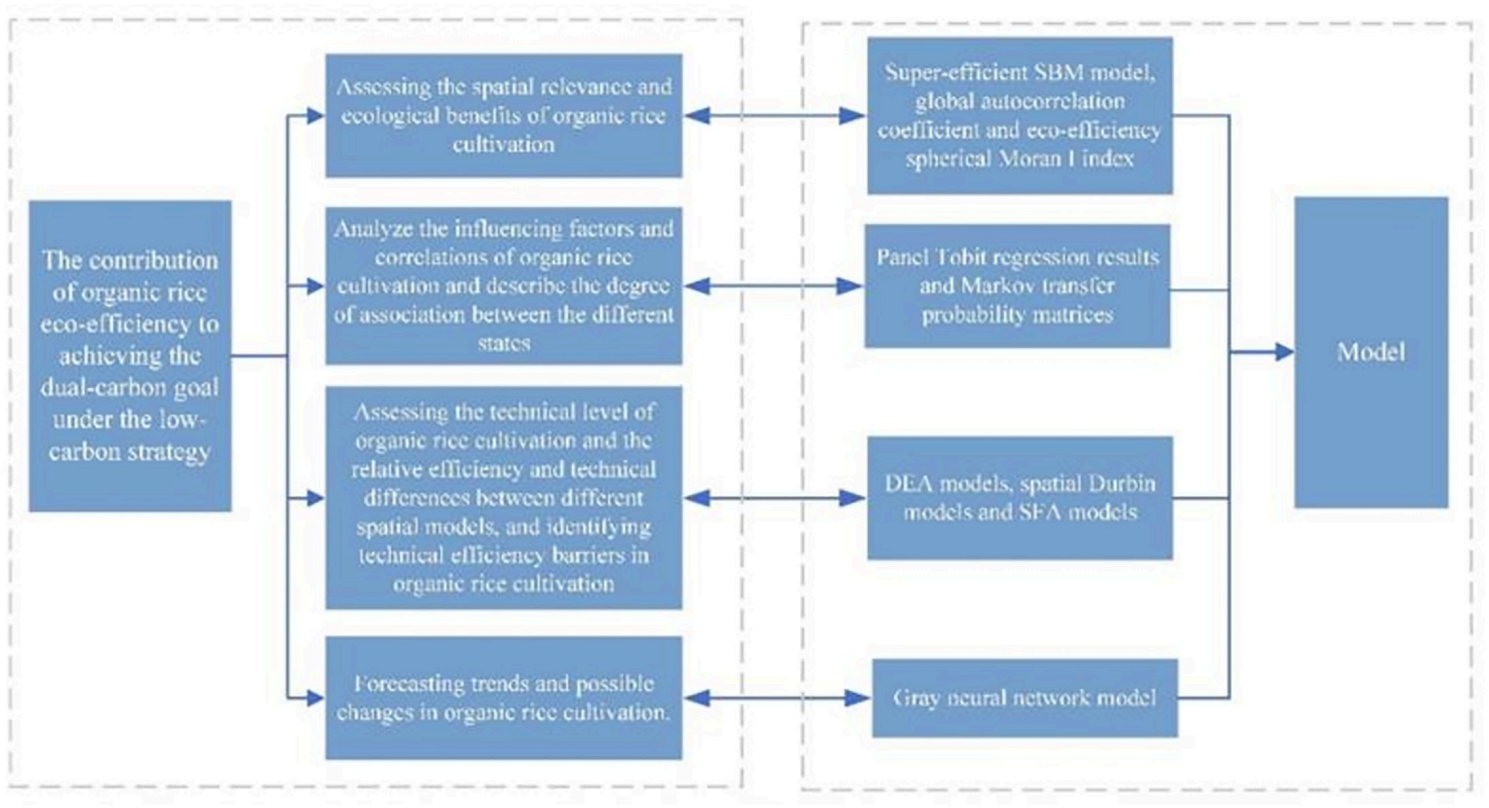
Model roadmap.

The main differences between organic rice and conventional rice include the following aspects: first, pesticide and fertilizer use, organic rice prohibits the use of chemically synthesized pesticides and fertilizers, and generally pays more attention to maintaining ecosystem complexity and stability in farm and crop management, thus increasing the potential for carbon reduction [37]. Conventional rice farming uses chemical pesticides and fertilizers to increase yields and control pests and diseases. The second is ecological environment protection. Organic agriculture can promote the survival and reproduction of natural enemies and beneficial insects by providing biodiversity habitats, thus reducing the dependence on chemical pesticides, and thus achieving natural biological control [21]. The use of chemical pesticides and fertilizers in traditional rice cultivation can pollute the environment and pose potential risks to the ecosystem. Third, organic agriculture usually adopts management measures such as natural ecological cycle, biodiversity conservation and soil health to achieve carbon emission reduction [32–34]. In traditional rice cultivation, the heavy use of chemical fertilizers may lead to the deterioration of soil quality and imbalance of farmland ecosystems. Overall, organic rice farming emphasizes eco-friendliness and sustainability, while conventional rice farming focuses more on high yields and economic efficiency. The Fig 1 map was as follows.

In this paper, the ecological benefits of organic rice and conventional rice were measured and compared using the provincial panel data and a relaxation-based econometric model from 2011 to 2021 in three provinces of Northeast China (Liaoning, Jilin and Heilongjiang). We combine Super-SBM model, spatial Durbin model (SDM) and Grey neural network model into a model integration framework to provide multi-angle and multi-dimensional analysis methods. First, the Super-SBM model is used to assess the efficiency of organic rice production, helping us understand the current situation and potential room for improvement. Second, SDM reveals spatial correlations and geographical influences, helping us to understand the differences and characteristics between different regions. Finally, GDM is responsible for predicting future trends and helping us predict the direction of the organic rice industry. By combining these three models, researchers can comprehensively understand the current situation of organic rice production, influencing factors and future development trends, so as to formulate more effective strategies to promote the sustainable development of organic rice industry.

### 3.1 Data selection

Since rice planting affects many factors such as paddy soil, biodiversity, chemical pollution, water pollution, and rice yield, the selected index considers whether its ecological benefit is representative. On the basis of comprehensive literature, this paper selected rice yield, sown area and related environmental indicators as indicators to measure the ecological benefits of rice, reflecting the overall situation of the two rice planting methods. In the process of rice production, the inputs of fertilizer such as nitrogen fertilizer, phosphate fertilizer, compound fertilizer, rice seed, agricultural film, diesel oil, as well as herbicides, pesticides, fungicides and other inputs are mainly considered. These indexes reflect the changes of rice cultivation from various pollution conditions, yield to application amount from different angles. The data of rice yield and sown area were obtained from China Agricultural Statistical Yearbook. The data of agricultural inputs (nitrogen, phosphorus, potassium, fertilizers such as compound fertilizers, rice seeds, agricultural film, diesel oil, etc.) in the process of rice production are derived from the National Agricultural Cost Income Information Compilation and the China Agricultural Statistical Yearbook. Herbicide, insecticide, fungicide dosage refer to the relevant literature. The relevant data of rice fertility were obtained from China Meteorological Data Network.

Organic and conventional rice production are two different agricultural systems that involve the process of growing, managing and treating rice crops. The main differences between organic rice and conventional rice include the following aspects: first, pesticide and fertilizer use, organic rice prohibits the use of chemically synthesized pesticides and fertilizers, and generally pays more attention to maintaining ecosystem complexity and stability in farm and crop management, thus increasing the potential for carbon reduction [37]. Conventional rice farming uses chemical pesticides and fertilizers to increase yields and control pests and diseases. The second is ecological environment protection. Organic agriculture can promote the survival and reproduction of natural enemies and beneficial insects by providing biodiversity habitats, thus reducing the dependence on chemical pesticides, and thus achieving natural biological control [21]. The use of chemical pesticides and fertilizers in traditional rice cultivation can pollute the environment and pose potential risks to the ecosystem. Third, organic agriculture usually adopts management measures such as natural ecological cycle, biodiversity conservation and soil health to achieve carbon emission reduction [32–34]. Studies have shown that organic agriculture can promote the accumulation of soil organic matter, improve soil fertility and water retention, thereby combating soil erosion and improving soil quality [46–48]. In traditional rice cultivation, the heavy use of chemical fertilizers may lead to the deterioration of soil quality and imbalance of farmland ecosystems. Overall, organic rice farming emphasizes eco-friendliness and sustainability, while conventional rice farming focuses more on high yields and economic efficiency.

### 3.2 Data processing

In the process of data processing, the Super-SBM model is used to eliminate the imperfect competition factors in the production process to better evaluate the ecological benefits. Although the traditional data envelope analysis model has a maximum efficiency value of 1, it is difficult to distinguish the efficiency difference of multiple DUS, and there are limitations in selecting regression model to analyze the influencing factors. Therefore, this paper chooses the superefficient slack-based measure model (super-SBM), which can further distinguish the efficiency degree of different DUS and overcome the above problems. Unlike traditional data envelopment analysis models, the optimal solution based on the relaxation measure model is dimensionless and allows the efficiency value of the relaxation measure model to be greater than 1 [70–72].

Suppose there are n decision units (*DMU*_*j*_ (*j*= 1, 2, …, *n*) in the organic rice ecosystem, and each decision unit contains m (*i* = 1, 2, …, *m*) production input factors, expected output factors and s (*r* = 1, 2, …, *s*) non-expected output factors. *DUM* to be evaluated is denoted *DMU*_*j*_ (*j* = 1, 2, …, *n*), let *x*_*ij*_ be the input *i* of the *j* th *DUM* and *y*_*rj*_ be the output *r* of the *j* th *DUM*.

The efficiency is:

$$P(x_{0},y_{0})=\{\left.\left(\bar{x},\bar{y}\right)\right|\bar{x}>\sum_{\text{k}=1,\neq 0}^{K}\lambda_{k}x_{k},\bar{y}\leq \sum_{\text{k}=1,\neq 0}^{K}\lambda_{k}x_{k},\bar{y}\geq 0,\lambda \geq 0\}$$ (1)

From this, the Super-SB model can be defined as:

$$\begin{array}{l} \text{min}\;\delta =\frac{\frac{1}{N}\sum_{n=1}^{N}\frac{{\bar{x}}_{n}}{x_{n0}}}{\frac{1}{M}\sum_{m=1}^{M}\frac{{\bar{y}}_{m}}{y_{m0}}}\\ s.t.\;\bar{x}\geq \sum_{k=1,\neq 0}^{K}x_{k}\lambda_{k}\\ \bar{y}\geq \sum_{k=1,\neq 0}^{K}y_{k}\lambda_{k}\\ \bar{x}\geq x_{0},\bar{y}\leq y_{0}\\ \sum_{k=1,\neq 0}^{K}\lambda_{k}=1\\ \bar{y}\geq 0,\lambda \geq 0 \end{array}$$ (2)

Then, with the help of the spatial Durbin model, the spatial correlation and spatial spillover effect of organic rice transformation in different regions were revealed. The spatial Durbin model is a statistical model commonly used to analyze spatial autocorrelation and spatial heterogeneity present in spatial data [72, 73]. Spatial Dubin models can be used to account for these spatial features to model and analyze space data more accurately [74]. Additionally, it is essential to note that the Spatial Durbin Model (SDM) is dynamic in nature, allowing for a comprehensive examination of the temporal evolution of spatial correlation and spatial spillover effects associated with organic rice conversion across diverse regions. Its expression is:

$$F_{it}=\beta x_{it}+\rho \sum_{j=1}^{n}W_{ij}F_{it}+\varphi \sum_{j=1}^{n}W_{ij}x_{it}+\mu_{i}+\nu_{t}+\varepsilon_{it}$$ (3)

where *F*_*it*_ is the observed value of the explanatory variable; *β* is the regression coefficient of the explanatory variable; *x*_*it*_ is the vector of influencing factors; *ρ* is the spatial regression coefficient of the explanatory variable; *φ* is the spatial regression coefficient of the explanatory variable; *W*_*ij*_ is the null autoweight matrix, and the geographic neighborhood spatial matrix was used in the present study: *μ*_*i*_, and *ν*_*t*_ denote the individual causal effect and the time-fixed effect, respectively, and *ε*_*it*_ is the random error term.

Finally, the gray neural network model was used to predict the development trend and benefit changes of organic rice conversion, which provided decision support and policy recommendations for policy makers. In this paper, a coupled gray dynamic (1,1) and BP neural network model is used. Among them, the gray dynamic model is mainly based on differential equations and exponential functions in the gray system. The intermediate results are obtained by graying the original data and establishing gray differential equations to preprocess and model the original data.

The grey dynamic (1, 1) prediction model is as follows:

Construct the original series:

$$x^{\left(0\right)}=\left\{x^{\left(0\right)}\left(1\right),x^{\left(0\right)}\left(2\right),x^{\left(0\right)}\left(3\right),\ldots x^{\left(0\right)}\left(N\right)\right\}$$

From the *x*^(0)^-series, continuous data of different lengths can be selected as subseries, and the steps for building the grey dynamic (1, 1) model for the subseries can be summarized as follows:

1. Determine any subdata sequence
$$x_{i}^{\left(0\right)}=\left[x^{\left(0\right)}\left(1\right),x^{\left(0\right)}\left(2\right),\ldots x^{\left(0\right)}\left(n\right)\right]$$
2. Sum the original series (1) once to obtain:
$$x^{\left(1\right)}=\left\{x^{\left(1\right)}\left(1\right),x^{\left(1\right)}\left(2\right),x^{\left(1\right)}\left(3\right),\ldots x^{\left(1\right)}\left(N\right)\right\}$$
  where $x^{\left(1\right)}\left(t\right)=\sum_{k=1}^{t}x^{\left(0\right)}\left(k\right)$
3. Construct the accumulation matrix B with the constant term vector
$$\left[\begin{array}{c} \begin{array}{cc} -\frac{1}{2}\left(x^{\left(1\right)}\left(1\right)\text{,}x^{\left(1\right)}\left(2\right)\right) & 1 \end{array}\\ \begin{array}{c} \begin{array}{cc} -\frac{1}{2}\left(x^{\left(1\right)}\left(2\right)\text{,}x^{\left(1\right)}\left(3\right)\right) & 1 \end{array}\\ \begin{array}{cc} \vdots & \vdots \end{array}\\ \begin{array}{cc} -\frac{1}{2}\left(x^{\left(1\right)}\left(N-1\right)\text{,}x^{\left(1\right)}\left(N\right)\right) & 1 \end{array} \end{array} \end{array}\right]$$
$$Y_{N}={\left[x_{1}^{\left(0\right)}\left(2\right)\text{,}x_{1}^{\left(0\right)}\left(3\right)\text{,}\cdots x_{1}^{\left(0\right)}\left(N\right)\right]}_{T}$$
4. Apply the least squares method to solve for the parameters:
$$â={\left[a,u\right]}^{T}={\left(B^{T}B\right)}^{-1}B^{T}Y_{N}$$
5. Bringing the grey parameters into the time function, calculate the difference between *x*^(0)^(*t*) and *x*^(0)^(*t*), *q*^(0)^(*t*) and the relative error: e (*t*):
$$\left.x^{\hat{\left(1\right)}}\left(t+1\right)=x^{\left(0\right)}\left(1\right)-\frac{u}{a}\right)e^{-at}+\frac{u}{a},$$
$$x^{\hat{\left(0\right)}}\left(t+1\right)=-a\left(x^{\left(0\right)}\left(1\right)-\frac{u}{a}\right)e^{-at},$$
$$x^{\hat{\left(0\right)}}\left(t+1\right)=x^{\left(1\right)}\left(t+1\right)-x^{\left(1\right)}\left(t\right)$$
$$q^{\left(1\right)}\left(t\right)=x^{\left(0\right)}\left(t\right)-x^{\left(0\right)}\left(t\right)$$
$$e\left(t\right)=q^{\left(0\right)}\left(t\right)/x^{\left(0\right)}\left(t\right)$$

Based on the prediction using the grey dynamic (1, 1) model, the accuracy of the prediction results is also diagnosed and judged. The reliability of the model is diagnosed using the posterior difference, and the observed data deviation s1 and the residual of the deviation s2 are calculated as follows:

s12=∑t=1m(x(0)(t)-x-(0)(t))2s22=1m-1∑t=1m-1(q(0)(t)-q-(0)(t)))2

The posterior test ratio and the small error probability are calculated as

p={|q(0)(t)-q-(0)|<0.6745s1}

The diagnostic criteria for the predicted outcomes are shown in Table 1:

**Table 1:** Diagnostic criteria for grey dynamic (1, 1).

C	P	Diagnosis result	Accuracy level
<0.35	>0.95	Good	Grade 1
<0.50	>0.80	Qualified	Grade 2
<0.65	>0.70	Barely qualified	Grade 3
> = 0.65	< = 0.70	Unqualified	Grade 4

BP Neural Network (Backpropagation Neural Network) is a common artificial neural network (ANN) architecture for supervised learning tasks. It is a multi-layer feed-forward neural network that typically includes an input layer, a hidden layer, and an output layer. The training process of BP neural network uses the backpropagation algorithm, a gradient descent optimization algorithm that is used to adjust the weights in the network to minimize the error between the predicted outputs and the actual outputs. The algorithm calculation steps are as follows:

(i)Data normalization process. Normalize all sample data by premnmx() function;(ii)Initialize the weights and bias of the neural network. The initial weights and biases are set randomly, the sample pattern counter and training times counter are set to 1, the error is set to 0, the learning rate is set to a decimal within 0~1, and the accuracy achieved after network training is set to a positive decimal; (iii) Pass the input data through the input layer of the network to the hidden layer, and then to the output layer. In each neuron, the output is calculated by weighted sum and activation function. (iv) Compare the network output with the target value and calculate the value of the loss function. Commonly used loss functions include mean square error (MSE) and cross-entropy loss function. (v) Based on the value of the loss function, backpropagate from the output layer to the hidden layer, calculate the error of each neuron, and adjust the weights and bias according to the error. (vi) Using gradient descent or other optimization algorithms, update the weights and bias of each neuron in the network according to the gradient of the error. (vii) Perform forward propagation and backpropagation iteratively until a predetermined stopping condition is reached, such as the maximum number of iterations is reached or the error is less than a certain threshold. (viii) Use the trained network to make predictions on new input data and output the results.

Grey Neural Network (GNN) usually refers to a model that combines gray theory and neural networks. Gray theory is a mathematical tool for dealing with systems with incomplete information, uncertainty and ambiguity. The purpose of combining neural networks with gray theory is to improve the modeling of uncertainty problems. The specific steps are:

(i) Predict the time series using the GM(1,1) model to obtain $\hat{X}(t)$ (ii)Build a neural network model. The network has three layers: input layer, hidden layer and output layer. (iii)The BP neural network is trained using the prediction error of the GM(1,1) model as input and the difference between the actual observation and the prediction of the GM(1,1) model as target output. (iv) Combine the predicted values of the GM(1,1) model with the predicted values of the BP neural network to get the prediction results of the final gray neural network model.

## 5 Results

### 5.1 Regression analysis

Organic rice production methods are more environmentally friendly and sustainable, whereas conventional rice production methods usually rely on the use of much chemical fertilizers and pesticides, which leads to environmental pollution and damage to the land ecosystem. Therefore, this paper intends to use data analysis to further determine whether organic rice cultivation can provide advantages in terms of eco-efficiency and carbon emissions.

The global autocorrelation coefficient is a statistic used to assess whether geographic phenomena are spatially autocorrelated; that is, the values of geographic phenomena that are close to each other affect or are similar to each other. Here, the autocorrelation coefficient of organic rice cultivation was calculated to determine whether organic rice cultivation is spatially autocorrelated, and Moran’s I statistic was used to measure the degree of correlation. Detailed information on the autocorrelation coefficient of organic rice cultivation, including Moran’s I statistic, the Z value and the P value for each year, is given in Table 2.

**Table 2:** Global autocorrelation coefficient of organic rice cultivation.

Year	2011	2012	2013	2014	2015	2016	2017	2018	2019	2020	2021
Moran’s	0.325	-0.085	0.152	0.241	0.264	0.168	0.362	0.248	0.954	0.249	0.254
Z-value	3.784	-1.56	3.026	3.415	3.054	4.265	4.051	2.265	2.365	2.035	2.795
P-value	0.002	0.003	0.065	0.004	0.006	0.001	0.002	0.034	0.041	0.24	0.009

According to Table 2, the Moran’s I statistic for organic rice cultivation is positive (0.152 to 0.362) in both 2011–2013 and 2015–2016, which indicates that there is a significant spatial autocorrelation of organic rice cultivation between these years, i.e., cultivation has internal effects or is similar between neighbouring geographical areas. However, the values of Moran’s I statistic in 2012 were largely negative, which implies that organic rice cultivation was not spatially significantly autocorrelated or showed spatial dispersion in these years.

In addition, Table 2 provides more intuitive information about the Z value, which is a standardized value that divides the Moran’s I statistic by the expected standard deviation, and the P value, which is the significance level under the null hypothesis. Taking 2011 as an example, the Z value of 3.784 implies that the hypothesis that organic rice cultivation is spatially autocorrelated can be rejected at the 99% confidence level, while the P value of 0.002 indicates very high significance.

In summary, this study found that organic rice planting has significant spatial autocorrelation in some years, indicating that organic rice planting has obvious spatial clustering characteristics. This may be due to terrain [75] and climatic conditions [76, 77]. At the same time, environmental pressure [78], cultivation conditions [79], market demand and economic cost [80] may also have an impact on the spatial distribution of organic rice cultivation.

The descriptive input‒output statistics of organic rice cultivation include five variables, where the output elements include GDP (billion yuan) and carbon emissions (million tons), and the input elements of capital stock (billion yuan), labour (10,000 people), and energy consumption (million tons of standard coal). Research has shown that organic farming can reduce reliance on external or synthetic inputs compared to conventional crop production methods [40, 81]. The mean, standard deviation, minimum, maximum and average annual growth rates (in percentage terms) of these variables are given in Table 3. From the perspective of output factors, the mean value of GDP for organic rice cultivation is 892.426 billion yuan, the standard deviation is 983.426 billion yuan, the minimum value is 17.244 billion yuan, and the maximum value is 694.510 billion yuan. A combination of the above data shows that organic rice cultivation can create great economic value, but there are substantial differences between different regions or years. In addition, carbon emissions are an important environmental issue. Table 3 shows that the average value of carbon emissions from organic rice cultivation is 206,452,100 tons, the standard deviation is 170,521,400 tons, the minimum value is 5,604,200 tons, and the maximum value is 86,542,200 tons. The carbon emissions of organic rice cultivation fluctuate widely and vary greatly from year to year. This indicates the need for measures to reduce carbon emissions from organic rice cultivation to achieve sustainable development.

**Table 3:** Descriptive statistics of input and output of organic rice cultivation.

Variable		Unit	Mean	Standard deviation	Min	Max	Annual average (%)
Output	GDP	billion yuan	8924.26	9834.26	172.44	69452.1	9.64
	Carbon Emissions	of million of tons	20645.21	17052.14	560.42	86542.12	6.23
Input	Capital stock	billion yuan	20144.98	25136.19	360.98	165425.2	14.5
	Labour	million	2516.21	1825.42	245.18	6826.14	2.06
	Energy consumption	Ten thousand tons of standard coal	9948.12	7826.43	310.26	44026.22	7.52

In terms of input factors, capital stock is an important investment in organic rice cultivation, which includes investment in land, equipment, and facilities. The mean, standard deviation, minimum and maximum values of capital stock for organic rice cultivation are given in Table 3. The results show that the average capital stock is 201.4498 billion yuan, the standard deviation is 2,513.619 billion yuan, the minimum value is 36.098 billion yuan, and the maximum value is 165.425.22 billion yuan. These results indicate that the scale of investment in organic rice cultivation is large but varies greatly between regions and between periods. Labour is also another important input for organic rice cultivation; Table 3 gives the mean, standard deviation, minimum and maximum values of the labour force for organic rice cultivation. The mean labour force is 25,162,100, the standard deviation is 18,254,200, the minimum value is 2,451,800, and the maximum value is 68,261,400. Similarly, there is a large variation in the labour force for organic rice cultivation. The last input element is the amount of energy consumption. Table 3 provides the mean, standard deviation, minimum and maximum values of energy consumption for organic rice cultivation. The results show that there are large fluctuations in the energy consumption of organic rice cultivation, with a mean value of 99,481,200 tons of standard coal, a standard deviation of 78,264,300 tons of standard coal, a minimum value of 3,102,600 tons of standard coal, and a maximum value of 44,026,200 tons of standard coal. The large variation in energy consumption between different regions and between different periods also requires measures to promote the low-carbon development of organic rice cultivation [82].

By combining the above results, it can be found that overall, the input‒output ratio of organic rice cultivation is high. First, an observation of the relationship between output index GDP and input index capital stock shows that the average annual output GDP basically reaches 40% of the input index capital stock, which indicates that organic rice cultivation has high economic benefits. Studies by Acs et al. have also found that organic farming is generally more profitable than conventional farming [83]. Second, by observing the relationship between output index GDP and output index carbon emission, we can learn that the average carbon emission of 10,000 Yuan GDP is only 6.23 tons. This shows that organic rice farming not only brings economic benefits, but also has a positive impact on the environment, so organic agriculture has the potential to protect the environment, improve ecosystem health and sustainable agricultural production [31]. Finally, analysing the growth rates of input and output factors, we find that the input growth rate is significantly higher than the output growth rate (except for the capital input growth rate), which indicates that the organic rice industry is in the stage of incremental economies of scale, and the higher capital input growth rate may be the reason why the organic rice industry has a high return on assets.

The global Moran’s I index of the eco-efficiency of organic rice cultivation is a measure of the eco-efficiency of organic rice cultivation on a global scale, and its value ranges from 0 to 1. The higher the value is, the higher the eco-efficiency of organic rice cultivation, and the more obvious its role in promoting the achievement of the dual carbon goal. The global Moran’s I index of the eco-efficiency of organic rice cultivation is shown in Table 4. The p values of Moran’s I index were all less than 0.05, indicating that the global Moran’s I index of the ecological efficiency of organic rice cultivation was significant from 2011 to 2021 and peaked in 2019.

**Table 4:** Ecological efficiency of organic rice cultivation global Moran’s I index.

Year	Moran’s I	Z Value	P Value
2009	0.3265	6.9254	0.001
2010	0.0415	3.0625	0.0051
2011	0.2685	8.0254	0.0002
2012	0.4025	11.2655	0.0006
2013	0.2351	9.0256	0
2014	0.1692	5.4258	0.0045
2015	0.0485	3.0265	0.0006
2016	0.2485	13.1452	0.0014
2017	0.2369	7.6425	0.0024
2018	0.3548	3.0256	0.0031
2019	0.5128	2.0654	0.0012
2020	0.4112	1.9546	0.0009
2021	0.3362	7.9956	0.0007

From the regression results in Table 5, this paper can conclude that farmers’ disposable income is an important factor contributing to the increase in sustainable development efficiency via organic rice cultivation and conventional cultivation. When farmers’ disposable income increases, both organic rice cultivation and conventional cultivation will significantly increase sustainable development efficiency. In contrast, the area of natural disasters (refers to the total area hit by natural disasters) and the area of disaster (refers to the number of acres of crops that were hit by natural disasters) are negative factors for sustainable development efficiency; these factors affect both organic rice cultivation and conventional cultivation. In addition, the increase in population significantly contributes to sustainable development efficiency in organic rice-growing areas, while the effect of population increase on sustainable development efficiency is not significant in conventional growing areas. The increase in GDP has a significant decreasing effect on both rice-growing areas, which may be due to the decrease in the importance of the primary sector when GDP increases.

**Table 5:** Factors affecting the efficiency of organic rice cultivation and conventional cultivation on sustainable development.

	Organic rice-growing area 1	Organic rice-growing area 2	Conventional planting area 1	Conventional planting area 2
Disposable income of farmers (yuan)	0.065***(0.023)	0.069**(0.021)	0.074**(0.011)	0.058***(0.015)
Area of natural disasters (thousand hm2)	-0.036***(0.005)	-0.026***(0.008)	-0.022***(0.007)	0.0036***(0.006)
Natural disaster area (thousand hm2)	-0.071**(0.018)	-0.057(0.128)	-0.058**(0.051)	-0.015**(0.018)
Population (10,000 people)	0.162***(0.049)	0.065*(0.098)	0.029**(0.0110)	0.0058***(0.006)
GDP (million yuan)	-0.00026	-0.00238	-0.190**(0.047)	-0.00346
Urbanization rate (%)	0.022*(0.064)	0.026*(0.087)	-0.006**(0.042)	-0.00332
Constant	-0.095(0.168)	-0.072(0.241)	-0.049**(0.028)	-0.047(0.195)

Note: P values in parentheses,

*, **, *** represent significance at the 10%, 5%, and 1% levels, respectively.

In particular, the increase in urbanization rate will significantly increase the planting area of organic rice, but significantly reduce the planting area of conventional rice, suggesting that the increase in urbanization rate will promote the planting of organic rice instead of conventional rice, which may be due to two reasons:

First, the process of urbanization has promoted the transformation and high-quality development of agriculture. Research has shown that urbanization can have a significant impact on rural development by facilitating information exchange [84]. More recently, empirical evidence from China also shows that urbanization can have a positive impact on agricultural production by freeing up rural land for agriculture, facilitating large-scale agricultural development, and promoting environmental protection [85]. These findings highlight the interrelationship between urbanization and agriculture and the potential opportunities of urbanization for agricultural development in rural areas.

Second, urban residents have higher requirements for the consumption quality of agricultural products. Studies have shown that urban areas are important markets for agricultural products and that urbanization can stimulate rural economies and farmer incomes by increasing the demand for natural resources [86]. Dorosh and Thurlow’s research found that urban growth leads to higher demand for agricultural products. Especially in areas where emerging middle class consumers are demanding diverse, high quality and safe products, the beginning of supermarkets increased the circulation of agricultural products and services, thus contributing to the growth of agriculture [87].

After the regression, this paper synthesizes the efficiency concept through a Markov transfer probability matrix of organic rice cultivation from 2009–2021, a DEA model efficiency comparison of current, overall and frontier technologies for organic rice cultivation, and a spatial Markov transfer probability matrix of organic rice cultivation from 2009–2021. Markov transition probability matrix is a mathematical tool used to describe the state transition probability in Markov chains. In a Markov chain, the future state of a system depends only on its current state, not on its past state. This property is called "memoryless" or "Markov". Markov transfer probability matrix has been applied in various fields, such as statistics, economics, physics, bioinformatics and so on. It is used to predict the evolution of system states, analyze stability, and conduct long-term behavior studies.

According to Table 6, a shift in organic rice cultivation can be seen. The probability matrix shows that among the four planting states, the fourth state has the highest probability (maximum 0.9041): 90% of the organic rice planting will remain as it is. The third state has the second highest probability (maximum 0.7052): 70% of the organic rice planting will remain as it is, which is related to the grower’s strategy. The second state has the third highest probability (maximum 0.5214): the second state ranked third (with a maximum of 0.5214), meaning that approximately half of the organic rice cultivation would continue as it was. Finally, the first state ranked last with a probability of 0.2695, implying that there was little improvement or innovation in organic rice cultivation, mainly due to financial reasons. A combination of the above results shows that organic rice cultivation has a positive feedback effect, where the better the cultivation status is, the more organic rice cultivation is driven.

**Table 6:** Markov shift probability matrix for organic rice cultivation, 2009–2021.

t\t+1	n	1	2	3	4
1	934	0.2695	0.3015	0.0094	0.0074
2	922	0.1752	0.5214	0.3015	0.0005
3	923	0.0045	0.2016	0.7052	0.2016
4	928	0.0025	0.0062	0.3015	0.9041

Table 7 depicts the data envelopment analysis model efficiency of current, overall and frontier technologies for organic rice cultivation. The conclusion shows that among these technologies, overall, the frontier technology Do has the highest efficiency, followed by the current technology Dt and finally the overall average Dg. Further analysis shows that the current technology has developed at a high level, but there is still much room for improvement. In addition, the coefficient of technological growth has become higher overall in recent years as time progresses, suggesting that improvements and innovations in organic rice cultivation technology are key to improving efficiency.

**Table 7:** Comparison of DEA model efficiency of current, overall, and frontier technologies for organic rice cultivation.

Year	$\vec{D^{t}}$	$\vec{D^{o}}$	$\vec{D^{G}}$	Year	$\vec{D^{t}}$	$\vec{D^{o}}$	$\vec{D^{G}}$
2000	0.2654	0.4025	0.3652	2011	0.3026	0.4265	0.2223
2001	0.3011	0.3015	0.2552	2012	0.4025	0.9254	0.4165
2002	0.3425	0.4075	0.1125	2013	0.0625	0.4155	0.4152
2003	0.0215	0.3625	0.4452	2014	0.5412	0.3624	0.5952
2004	0.4156	0.7954	0.4652	2015	0.3362	0.8145	0.4412
2005	0.2651	0.4521	0.2695	2016	0.8452	0.9243	0.4162
2006	0.3026	0.3625	0.8845	2017	0.4152	0.7156	0.3552
2007	0.4581	0.5210	0.4415	2018	0.3362	0.6625	0.2458
2008	0.6252	0.4215	0.3625	2019	0.4155	0.4415	0.3621
2009	0.7185	0.3621	0.5425	2020	0.3321	0.9246	0.3662
2010	0.2654	0.7251	0.6251	2021	0.9541	0.7256	0.3585

Table 8 refers to the method of the reference [25] to test the stochastic frontier model in two ways: firstly, the production function form is tested to determine whether it is correct to adopt the form of the transcendental logarithmic production function containing the proxy variable t for technological progress. Hypothesis H1: there is no interaction between the variables, i.e., the coefficient of the cross term is 0; Hypothesis H2: there is no technological progress, i.e., the coefficient of the polynomial containing the variable t is 0. The second is to test the inefficiency function by constructing Hypotheses H3, H4, and H5 assuming, respectively, that the technological inefficiency index (*μ*), the rate of change of the technical efficiency (*η*), and the technical inefficiency term as a proportion of the composite disturbance term (*δ*) is 0. The test results are shown in Table 2, all of which strictly reject the original hypotheses, indicating that the use of the transcendental logarithmic production function is in line with the reality of the study, that the technical inefficiency index does not obey the semimodal normal distribution, that the technical efficiency is time-varying, and that there is indeed a technical inefficiency.

**Table 8:** Results of hypothesis testing of stochastic frontier model.

Hypothesis	Degree of freedom	LR	
H1: *β*_*ii*_ = *β*_*ik*_ = $\beta_{t^{2}}$ = *β*_*it*_ = 0	15	1.7962***	Rejection
H2: *β*_*t*_ = $\beta_{t^{2}}$ = *β*_*it*_ = 0	6	31.41***	Rejection
H3: *μ* = 0	1	83.21***	Rejection
H4: *η* = 0	1	98.39***	Rejection
H5: *μ* = *η* = γ = 0	3	72.69***	Rejection

Table 9 The regression results are shown in Table 3. x1 denotes capital stock inputs, x2 denotes labor inputs, and x3 energy consumption inputs. The value of γ is 0.6429, indicating that 64.29% of the compound error term comes from technical inefficiency. η is 0.4137 and significant at the 5% level, indicating that the technical efficiency shows a progressive trend.

**Table 9:** Coefficient estimation results of stochastic frontier model.

Variable	Parameter to be estimated	Coefficient	Standard error
t	β1	0.4379	0.035
Lnx1	β2	0.4962***	0.014
Lnx2	β3	0.3148***	0.049
Lnx3	β4	-0.0147**	0.011
t2	β5	0.0351***	0.027
Lnx1^2^	β6	0.4269***	0.096
Lnx2^2^	β7	0.7643***	0.013
Lnx3^2^	β8	-0.1597**	0.041
tLnx1	β9	-0.3179	0.083
tLnx2	β10	-0.4792	0.047
tLnx3	β11	0.1398	0.029
Lnx1 lnx2	β12	0.5984**	0.091
Lnx1 lnx3	β13	-0.1743*	0.007
Lnx2 lnx3	β13	-0.1291**	0.016
cons	β0	3.7643	5.018
μ		2.1687**	4.782
η		0.4137**	0.014
σ^2^		0.2143	0.012
γ		0.6429	0.014

Note:

* P<0.10,

** P<0.05,

*** P<0.01.

In terms of the coefficients of the input factors, the coefficient of capital stock input is the largest at 0.4962, followed by labor input with a coefficient of 0.3148. The coefficients of the primary term and the squared term of the above two factor inputs are significantly positive at the 1% level, which indicates that the increase of inputs in terms of capital stock and labor can significantly contribute to the increase of output. The coefficient of the primary term of energy consumption is -0.0147, which is significantly negative at the 5% level and the coefficient of the squared term of service inputs is also significantly negative, indicating that energy consumption in the study sample has a significant dampening effect on output increase. This problem may be attributed to the fact that excessive energy consumption directly affects the sustainability and environmental friendliness of organic farming and leads to over-dependence on energy. From the cross term coefficients, the cross term coefficients of capital stock inputs and labor inputs are significantly positive, which indicates that the two input factors are mutually reinforcing. This implies that increasing capital stock along with labor inputs can be more effective in improving the output and agricultural productivity of organic rice. The coefficients of the cross terms of capital stock and energy-consuming inputs, and labor inputs and energy-consuming inputs are all significantly negative, which indicates that the current coordination between the factors and energy-consuming inputs is not high, and it will have some inhibitory effects.

From the above analysis, it can be seen that there is currently an excessive energy dependence in organic rice cultivation leading to unsustainable development of organic rice. The results of the study show that the coordination between energy consumption and other input factors is not high, which indicates that the overall optimization and synergy between energy consumption and other input factors need to be improved in the current agricultural practice. Meanwhile, encouraging farmers to introduce advanced agricultural technology and equipment to improve the effectiveness of labor is also one of the directions for the development of organic rice cultivation. Therefore, the overall optimization of the combination of input factors and the coordination between them should be improved to achieve more efficient organic rice cultivation.

Table 10 shows the shifts in organic rice cultivation in different spatial regions. The second state has the highest probability in spatial region 1 (maximum 0.6254), which is because growers in this region prefer to maintain their original cultivation strategy. In spatial region 2, cutting-edge technologies are more widely used, so the fourth state has the highest probability (maximum 0.9933): organic rice cultivation is more technologically advanced. In spatial region 3, due to poorer environmental quality, the probability of the first state is higher (maximum 0.5026): organic rice cultivation may adopt more environmentally friendly cultivation methods. In spatial region 4, the probability of the second state is the highest (maximum 0.8012), indicating that growers in this region prefer to keep their original cultivation strategy and adopt some more efficient cultivation techniques. Combining the above results, this paper shows that organic rice cultivation has a positive feedback effect: the better the planting state is, the more it will drive organic rice cultivation.

**Table 10:** Spatial Markov shift probability matrix for organic rice cultivation, 2009–2021.

Spatial lag	t\t+1	n	1	2	3	4
1	1	678	0.6254	0.3054	0.0062	0.1524
	2	421	0.2015	0.5214	0.0048	0.3625
	3	201	0.0023	0.3145	0.1425	0.4955
	4	65	0.01425	0.0241	0.1552	0.5162
2	1	152	0.5218	0.6254	0.2565	0.6029
	2	431	0.0062	0.4429	0.1144	0.7824
	3	405	0.0047	0.5932	0.9524	0.8145
	4	152	0.0001	0.6041	0.1152	0.9933
3	1	15	0.5026	0.2014	0.2004	0.4582
	2	99	0.0954	0.3026	0.0036	0.9246
	3	272	0.0262	0.0426	0.0042	0.8556
	4	130	0.0002	0.4814	0.0142	0.2651
4	1	6	0.2059	0.5241	0.6254	0.4157
	2	42	0.0841	0.8012	0.7152	0.3311
	3	182	0.0847	0.6231	0.4821	0.2514
	4	569	0.0042	0.5471	0.2695	0.3369

### 5.2 Spatial durbin model analysis

In this section, the dynamic spatial econometric model Spatial Durbin Model is used in this paper. The model takes into account not only the influence of the region’s own variables on ecological benefits, but also the influence of neighboring regions’ variables on ecological benefits. At the same time, the model introduces the Kalman filter variable to explain the estimation of unknown states, which may be related to spatial dynamics. With the Kalman filter, the model can better capture the unknown dynamic effects of spatial correlations, thus improving the ability to model spatial changes. The results of the spatial Durbin model are shown in Table 11 below.

**Table 11:** Results of the spatial Durbin model.

Models	Model1	Model2	Model3	Model4	Model5	Model6	Model7	Model8
	Baseline regression				Explanatory variables lagged by one period			
Spatial weight matrix	$W_{d}^{H}$	*W*_*e*_	$W_{ge}^{H}$	$W_{w}^{H}$	$W_{w}^{H}$	$W_{d}^{H}$	$W_{ge}^{H}$	$W_{w}^{H}$
P	0.3625^b^ (0.2013)	0.2265^a^ (0.0452)	0.1834^a^ (0.0655)	0.2985^a^ (0.0958)	0.2063^a^ (0.0459)	0.4825^a^ (0.0365)	0.5985^a^ (0.5985)	0.555^a^ (0.2795)
Interval discrimination	-0.3485 (0.12254)	-0.2552^b^ (0.0013)	-0.01625 (0.0123)	0.0265^c^ (0.04529)	-0.06245^a^ (0.2458)	-0.7695^a^ (0.1365)	-0.36625^a^ (0.2995)	-0.3555^b^ (0.20588)
STQ	-0.8625^a^ (0.2036)	-0.7825^a^ (0.09546)	-0.8495^a^ (0.1452)	-0.7065^a^ (0.2044)	-0.2849^a^ (0.2245)	-0.2265^a^ (0.1595)	-0.4825^a^ (0.4965)	-0.5825^b^ (0.6953)
OS	-0.02365 (0.0265)	-0.04265 (0.0958)	0.02658 (0.02652)	-0.04562 (0.01459)	-0.0652^a^ (0.0265)	-0.04555^a^ (0.2758)	-0.75985^a^ (0.4458)	0.3775^a^ (0.4443)
ES	-0.3695^a^ (0.0426)	-0.2256^a^ (0.0365)	-0.2695^a^ (0.0425)	-0.2499^a^ (0.004295)	0.4595 (0.01552)	-0.5956^b^ (0.04855)	-0.3555^a^ (0.03253)	-0.3625^a^ (0.2575)
UR	-0.4692^a^ (0.2215)	-0.4985^a^ (0.1125)	-0.6042^a^ (0.1409)	-0.6025^a^ (0.2154)	-0.22655^a^ (0.2066)	0.0425^a^ (0.04295)	-0.8525^a^ (0.2559)	-1.325^a^ (0.5554)
UR^2^	-0.4692^b^ (0.0263)	0.04925^a^ (0.01459)	0.5985^a^ (0.01958)	0.08845^a^ (0.04655)	0.0625^a^ (0.01254)	-0.9588^a^ (0.00452)	-0.5525^a^ (0.2558)	-0.7955^a^ (0.2544)
TI	0.0642^b^ (0.0045)	0.04595^a^ (0.00495)	0.04265^a^ (0.00695)	0.03451^a^ (0.00485)	0.04255^b^ (0.00785)	-0.3795^a^ (0.2985)	-0.355^a^ (0.0065)	-0.5725^b^ (0.553)
EE	0.2954^a^ (0.04252)	0.2236^a^ (0.01692)	0.2451^a^ (0.0499)	0.3695^a^ (0.0152)	0.2366^a^ (0.00459)	-1.355^a^ (0.2253)	-1.3625^a^ (0.2458)	0.5525^a^ (0.255)
w Interval discrimination	-0.4026^a^ (0.06245)	-0.2036^a^ (0.02384)	-0.0954^a^ (0.01295)	-0.09558^b^ (0.04525)	-0.9355^a^ (0.0655)	0.2958^a^ (0.2582)	-0.5525^a^ (0.2355)	-0.5525^b^ (0.2525)
w Kalman filter	0.5369 (0.2013)	1.2685^a^ (0.0185)	0.8695^a^ (0.2236)	0.8954^a^ (0.1952)	1.2655^b^ (0.3495)	-0.6951^a^ (0.01256)	-0.3625^a^ (0.2953)	-0.5525^a^ (0.2555)
wOS	0.3625^b^ (0.4406)	-0.03655 (0.06254)	-0.0459 (0.0485)	-0.04529^a^ (0.2955)	-0.04525^a^ (0.0652)	-0.1225^a^ (0.4265)	-0.955^a^ (0.5553)	0.59625^a^ (0.2783)
wES	-0.6629 (0.2695)	-0.2694^a^ (0.06245)	-0.3475^a^ (0.0654)	-0.2265^a^ (0.0625)	0.3625^b^ (0.2013)	-0.7925^a^ (0.1254)	0.3575^a^ (0.365)	-0.5955^a^ (0.4983)
wUR	2.0362^b^ (0.8953)	1.6245^a^ (0.2635)	0.7062^a^ (0.3062)	1.9522^a^ (0.4035)	-0.69585^a^ (0.2558)	-0.3672^a^ (0.2655)	-0.7825^a^ (0.2693)	-0.7625^a^ (0.2055)
wUR^2^	-0.3795^b^ (0.2265)	-0.1425^a^ (0.04655)	-0.2065^a^ (0.2013)	-0.2369^a^ (0.0065)	-0.3625^a^ (0.2758)	0.4855^a^ (0.14993)	-0.4485^a^ (0.8713)	-0.3625^c^ (0.2553)
wTI	0.1694^a^ (0.0492)	0.02654 (0.0125)	0.0695 (0.00452)	0.0425^b^ (0.0205)	0.1275^a^ (0.04525)	0.79855^a^ (0.2142)	-0.37955^a^ (0.20455)	-0.9525^a^ (0.2555)
wEE	0.4395^b^ (0.1895)	-0.3495 (0.03652)	-0.37649 (0.2013)	0.00299 (0.0896)	0.37955^b^ (0.1245)	-0.7825^a^ (0.9853)	-0.34455^a^ (0.2785)	-1.2225^a^ (0.2958)
Constant terms	0.03625^a^ (0.00125)	0.3798^a^ (0.00203)	0.04552^a^ (0.00425)	0.0362^a^ (0.004253)	0.04525^a^ (0.00123)	-0.3565^a^ (0.2485)	0.47285^a^ (0.4283)	-0.5525^a^ (0.2583)
Control variables and their spatial terms	Control	Control	Control	Control	Control	Control	Control	Control
Time fixed effects	Control	Control	Control	Control	Control	Control	Control	Control
Individual fixed effects	Control	Control	Control	Control	Control	Control	Control	Control
Wald test (SAR) (p value)	69.23 (0.000)	162.45 (0.000)	143.26 (0.000)	156.42 (0.002)	61.62 (0.000)	136.42 (0.002)	121.42 (0.000)	120.44 (0.000)
Wald test (SEM) (p value)	94.62 (0.000)	195.24 (0.000)	160.42 (0.000)	178.94 (0.000)	88.69 (0.000)	169.42 (0.000)	195.42 (0.000)	142.51 (0.000)
Wald test (parametric joint test) (P value)	1306.23 (0.000)	1625.34 (0.001)	1546.28 (0.000)	1594.23 (0.000)	1605.42 (0.000)	1504.26 (0.000)	1402.5 (0.000)	1395.44 (0.000)
Sample size	700	700	700	700	700	700	700	700

Based on the above model data, the results of the specific multivariate spatial Durbin models (Model 1—Model 8) can be seen, each controlling for individual fixed effects and time fixed effects and introducing various explanatory variables and their spatial terms. Models 1–8 correspond to different spatial weight matrices and introduce several explanatory variables and their spatial terms while controlling for individual fixed effects and time fixed effects.

The coefficient of P is significantly positive in both model 1 (P = 0.3625b, p value = 0.000) and model 8 (P = 0.555a, p value = 0.000), thus indicating a positive effect of P on the explanatory variables. The coefficient of interval discrimination (IS) is negative in both model 1 (IS = -0.3485, p value = 0.000) and model 8 (IS = -0.3555b, p value = 0.000), indicating that IS has a negative effect on the explanatory variables and that this effect is most significant in model 8. The coefficient of STQ is negative in both Model 1—Model 5 and Model 8, but this effect is not significant in all models except Model 3. The coefficient of OS is insignificant in both Model 2 (OS = -0.04265, p value = 0.06254) and Model 8 (OS = 0.59625a, p value = 0.2783), but in Model 5 (OS = -0.0652a, p value = 0.0265), there is a negative significant effect. The coefficients of the variable energy per symbol (ES) in models 1–5 are all negatively significant (e.g., the ES coefficient in model 4 is 0.3695a, p value 0.0152), which indicates that ES has a negative effect on the explanatory variables.

The variables UR and UR^2^ have different influences in the different models, but their main results indicate that the explanatory variables decrease significantly as UR and UR^2^ increase. The coefficient of TI is positive in all models 1–5 (e.g., the coefficient of TI in model 2 is 0.04595a with a p value of 0.00495), which indicates that TI has a positive influence on the explanatory variables. The coefficients of the variable economic return (EE) in models 1–5 are all positive (e.g., the coefficient of EE in model 1 is 0.2954a with a p value of 0.04252), which also indicates that EE has a positive effect on the explanatory variables.

Finally, while controlling for other variables, models 1–8 all aim to predict the explanatory variables. To check the quality of the models, the Wald test was also conducted. The results show that the joint test of the parameters of the models in this paper is highly significant (all p values are 0.000), which indicates that the models are very effective in explaining the explanatory variables. Moreover, this paper effectively explains the changes in the explained variables through the weight matrix, the explanatory variables and their spatial terms.

The above data analysis shows that the factors affecting the ecological efficiency of organic rice cultivation include various factors, such as the level of technology and environmental quality, and that technological innovation and improvement can improve the efficiency of organic rice cultivation, thus achieving both economic and ecological benefits while helping to achieve low-carbon goals [50].

### 5.3 Gray neural network model

The prediction of ecological benefit of organic rice is a complex task, which involves the interaction of many influencing factors, such as vegetation coverage rate, agricultural irrigation rate, fertilizer application amount, and agricultural film use amount. The traditional prediction model has some limitations in dealing with such nonlinear and unstable data. At the same time, there are often incomplete and uncertain data in the field of agriculture. Grey neural network models can provide more accurate and reliable predictions by filling in missing data and processing unstable data.

As can be seen from Table 12, the average relative error of the gray dynamic model from 2012 to 2021 is 1.53%, while the value of the gray neural network is only 0.52%, which indicates that the gray neural network has a lower average relative error relative to the gray dynamic model. Gray dynamic model is a method based on gray system theory, which is suitable for data of first-order dynamic nature, but may have some limitations when dealing with complex nonlinear data. The gray neural network model, on the other hand, combines the nonlinear fitting ability of neural networks, which can better adapt to and model complex data relationships and improve the accuracy and stability of prediction. Therefore the model can be used for practical prediction.

**Table 12:** Effectiveness of total agricultural carbon sink value prediction based on gray neural network modelling.

Year	Actual Value (10,000 tons)	Grey Dynamic Model Simulated Value (10,000 tons)	Relative Error of Grey Dynamic Model (%)	Grey Neural Network Simulated Value (10,000 tons)	Relative Error of Grey Neural Network Model (%)
2012	15783	15783	0	15783	0
2013	15762	15557	1.35	15725	0.23
2014	15642	16003	-2.31	15852	-1.34
2015	15488	15322	1.07	15479	0.06
2016	15416	15350	0.43	15365	0.33
2017	15292	15792	-3.27	15361	-0.45
2018	15076	15350	-1.82	15065	0.07
2019	15133	15038	0.63	15069	0.42
2020	14962	14675	1.92	14522	1.07
2021	14980	15125	-0.97	15080	-0.67

The Table 13 projections show that the vegetation cover in 2030 is 57.83% while in 2060 it is 70.61%. This indicates that the vegetation cover is expected to show an increasing trend in the coming decades. This may be a result of increased awareness of environmental protection and implementation of ecological restoration projects. Irrigated agriculture is 61.33% in 2030 compared to 66.47% in 2060, which is a smaller increase. This may be due to the impact of factors such as climate change and water scarcity, and the agricultural sector will focus more on improving irrigation efficiency and water conservation. In addition, improvement in agricultural irrigation technology and optimization of irrigation management will also contribute to the increase in irrigation rate. For the analysis of fertilizer use, the forecast results show that the use of fertilizer in 2030 is 41.73 million tons, while in 2060 it is 33.71 million tons. This implies that the demand for fertilizers in agricultural production will decline in the future. This may be due to the adoption of more organic and ecological farming methods in the agricultural sector as an alternative to the traditional application of large quantities of chemical fertilizers. Regarding the use of agricultural films the projections show that the use of agricultural films in 2030 is 2.13 million tons as compared to 1.71 million tons in 2060. This indicates that the demand for agricultural films may decrease in the future. In addition, the analysis on the value of agricultural output shows that the forecast results show that the total value of agricultural production in 2030 is 90,217,910,000 Yuan, while in 2060 it is 11,729,228,000 Yuan. This indicates that the total value of agricultural production is expected to increase in the future. This can be attributed to the effects of population growth and economic development, as well as the increase in agricultural production capacity as a result of modernization and technological advancement in agriculture. Finally, for the analysis of carbon sinks and emissions, the projections show that the total carbon sink value of agriculture in 2030 is 994.45 million tons compared to 910.86 million tons in 2060. In contrast, total carbon emissions from agriculture in 2030 are 804.62 million tons compared to 715.61 million tons in 2060. This suggests that in the future, the agricultural sector is likely to focus more on reducing carbon emissions and increasing carbon sinks in order to address climate change and environmental challenges.

**Table 13:** Gray neural network model prediction results.

Year	2030	2060
Vegetation Coverage (%)	57.83	70.61
Agricultural Irrigation Rate (%)	61.33	65.47
Fertilizer Usage (10,000 tons)	4173	3371
Agricultural Film Usage (10,000 tons)	213	171
Agricultural Output Value (Million yuan)	9021791	11729228
Agricultural Total Carbon Sequestration (10,000 tons)	99445	91086
Agricultural Total Carbon Emissions (10,000 tons)	80462	71561

### 5.4 Assessment of organic rice’s contribution to achieving the dual carbon goal

#### 5.4.1 Carbon emissions

Organic rice has lower carbon emissions than conventional rice. Established literature has demonstrated that organic agriculture reduces greenhouse gas emissions by increasing soil organic matter and optimizing agroecosystems to reduce gas emissions. According to relevant data, the carbon emissions of organic rice are reduced by approximately 20% compared to conventional rice. This is because organic agriculture focuses on not only economic benefits but also ecological benefits and does not use chemical fertilizers and pesticides, which improves the stability of the soil ecosystem and reduces ecological risks such as soil respiration surge, soil microbial base, and soil fertility, which in turn reduces carbon emissions.

#### 5.4.2 Analysis of the direct and indirect effects of carbon emissions

The purpose of this paper is to investigate the ecological efficiency of organic rice and conventional cultivation and their contributions to achieving the dual carbon goal under the low carbon strategy. The study uses eight models to assess the direct and indirect effects of organic rice and conventional farming on carbon emissions from different perspectives, as shown in Table 14.

**Table 14:** Direct and indirect effects of organic rice culture and conventional culture on carbon emissions.

Model		Model1	Model2	Model3	Model4	Model5	Model6	Model7	Model8
		Baseline regression				Explanatory variables lagged by one period			
Spatial weight matrix		$W_{d}^{H}$	*W*_*e*_	$W_{ge}^{H}$	$W_{w}^{H}$	$W_{w}^{H}$	$W_{d}^{H}$	$W_{ge}^{H}$	$W_{w}^{H}$
Direct effect	Interval discrimination	-0.03585^c^ (0.04254)	-0.02552^a^ (0.0063)	-0.0165 (0.0023)	0.0275^c^ (0.04558)	-0.06585^a^ (0.2248)	-0.3625^a^ (0.7865)	-0.35525^a^ (0.275)	-0.155^b^ (0.5588)
	Kalman filter	-0.9855^a^ (0.1436)	-0.9525^a^ (0.02546)	-0.8585^a^ (0.17552)	-0.7695^a^ (0.2494)	-0.2489^a^ (0.2245)	-0.2255^a^ (0.12235)	-0.6925^a^ (0.4362)	-0.7725^b^ (0062953)
	OS	-0.04865 (0.14455)	-0.04955 (0.0468)	-0.02658 (0.03652)	-0.04582 (0.01448)	-0.0782^a^ (0.0365)	-0.04955^a^ (0.3058)	-0.75485^a^ (0.4698)	0.4275^a^ (0.4953)
	ES	-0.3475^a^ (0.06956)	-0.2256^a^ (0.07955)	-0.2985^a^ (0.0425)	-0.3659^a^ (0.004485)	0.55 (0.01425)	-0.5956^b^ (0.04955)	-0.4955^a^ (0.01253)	-0.5625^a^ (0.2765)
	UR	-0.4365^a^ (0.12455)	-0.9585^a^ (0.2365)	-0.6252^a^ (0.1959)	-0.6255^a^ (0.2904)	-0.225^a^ (0.2095)	0.0445^a^ (0.07855)	-0.9925^a^ (0.2459)	-0.9525^a^ (0.2.63)
	UR^2^	-0.4892^a^ (0.1654)	0.05425^h^ (0.01958)	-0.1285^a^ (0.06258)	0.0825^a^ (0.04555)	0.0125^a^ (0.0148)	-0.0788^a^ (0.0047)	-0.4485^a^ (0.6358)	-0.8655^a^ (0.3624)
	TI	0.03552^a^ (0.00755)	0.04265^a^ (0.00695)	-0.05265^a^ (0.00795)	0.02251^a^ (0.00455)	-0.03355^b^ (0.0075)	-0.7695^a^ (0.3085)	-0.495^a^ (0.0075)	-0.4925^b^ (0.543)
	Economic return	0.3654^a^ (0.0246)	0.2686^a^ (0.0175)	0.8551^a^ (0.0699)	0.36925^a^ (0.0122)	0.256^a^ (0.00485)	-1.075^a^ (0.7453)	-1.4625^a^ (0.2258)	0.7125^a^ (0.885)
Indirect effect	Interval discrimination	-0.495^a^ (0.06485)	-0.2756^a^ (0.0295)	-0.0854^a^ (0.01245)	-0.0258^b^ (0.0454)	-0.5255^a^ (0.0955)	0.2548^a^ (0.382)	-0.3625^a^ (0.2355)	-0.7525^b^ (0.2525)
	Kalman filter	0.5395 (0.7598)	1.23655^a^ (0.0255)	0.6495^a^ (0.2356)	-0.9954^a^ (0.1972)	-0.2755^a^ (0.6895)	-0.8051^a^ (0.01556)	-0.9525^a^ (0.2033)	-0.7965^a^ (0.365)
	OS	0.3495 (0.1366)	-0.0355 (0.06584)	-0.049 (0.0448)	-0.05429^a^ (0.2955)	-0.06825^a^ (0.0492)	-0.1525^a^ (0.4365)	-0.955^a^ (0.5553)	0.955^a^ (0.6683)
	ES	-2.6259^a^ (0.5365)	-0.2655^a^ (0.04855)	-0.2275^a^ (0.0494)	-0.4455^a^ (0.0655)	-0.7525^b^ (0.2213)	-0.6925^a^ (0.1664)	0.4475^a^ (0.2265)	-0.9525^a^ (0.4542)
	UR	2.1452^b^ (1.633)	1.985^a^ (0.27585)	0.652^a^ (0.3452)	-0.9522^a^ (0.4955)	-0.70585^a^ (0.6958)	-0.7572^a^ (0.7955)	-0.8525^a^ (0.269)	-0.9348^a^ (0.7265)
	UR^2^	-0.4595^b^ (1.3665)	-0.135^a^ (0.04485)	-0.2415^a^ (0.2123)	-0.2769^a^ (0.0065)	-0.3485^a^ (0.3658)	0.4785^a^ (0.1556)	-0.4635^a^ (0.9373)	-0.2245^c^ (0.958)
	TI	0.3524^a^ (0.007582)	0.026485 (0.0025)	0.0748 (0.0044)	0.0325^b^ (0.0405)	0.1585^a^ (0.05525)	0.6955^a^ (0.4942)	-0.31255^a^ (0.2755)	-0.9345^a^ (0.278)
	Economic return	0.4895^b^ (0.19355)	-0.34588 (0.004252)	-0.3775 (0.2473)	0.00399 (0.0606)	0.49555^b^ (0.1785)	-0.6825^a^ (0.7553)	-0.2455^a^ (0.225)	-1.2425^a^ (0.293)

From the perspective of direct effects, organic rice has a certain degree of reduction in carbon emissions compared to conventional cultivation. The coefficient of Interval discrimination in model 3 is 0.0275, implying that IS emissions from organic rice cultivation are 0.0275 units less than those from conventional cultivation. The coefficient of Kalman filter in model 2 is -0.9525, implying that Kalman filter emissions from organic rice cultivation are 0.9525 units less than those from conventional cultivation. These coefficients show that the adoption of organic rice cultivation can reduce the direct effect of carbon emissions to some extent.

From the perspective of indirect effects, organic rice has a positive contribution to achieving the dual carbon goal. The coefficient of Kalman filter in model 1 is 0.5395, implying that the adoption of organic rice cultivation increases the likelihood of achieving the dual carbon goal. The coefficient of Economic return in model 1 is 0.4895, implying that organic rice cultivation contributes to increasing carbon neutrality and economic return. Although the coefficients of some variables are negative, the overall trend indicates that the adoption of organic rice can contribute to some extent to the achievement of the dual carbon goal. The results suggest that organic rice has the benefit of reducing direct carbon emissions compared to conventional cultivation while being able to contribute to some extent to the achievement of the dual carbon goal.

### 5.5 Analysis of economic benefits

By analyzing the economic benefits of organic rice, the government and relevant authorities can assess the profitability of growing organic rice, thereby making informed decisions and optimizing the use of resources. Meanwhile, understanding the market opportunities for organic rice can capture the growing consumer demand for organic products and provide better marketing strategies for organic rice. This provides an important scientific basis for promoting the sustainable development of organic agriculture.

Table 15 shows the average mu yield of organic and conventional rice production in Northeast China over the years. In 2012, the mu yield of organic rice was 437 kg/mu, which accounted for 64% of the mu yield of conventional rice. After 2018, the mu yield of organic rice was more than 600 kg/mu, which accounted for more than 80% of the mu yield of conventional rice. The results of the above study show that the mu yield of organic rice has increased year by year, but is lower than that of conventional rice, due to the prohibition of the use of substances such as chemically synthesized pesticides and fertilizers in organic agriculture, as well as the problems of insufficient soil fertility and inadequate control of pests, diseases, and weeds that existed in the early stages of organic conversion. However, over time, organic rice yields gradually approached those of conventional rice. After 2015, the percentage of organic rice yield relative to that of conventional rice began to increase, from 71% to 84%.

**Table 15:** Organic and conventionally cultivated rice yields in different years from 2012 to 2021.

Year	Organic Yield (kg/mu)	Conventional Yield (kg/mu)	Organic vs Conventional (kg/mu)	Organic Yield as Percentage of Conventional (%)
2012	437	684	-247	64
2013	455	708	-253	64
2014	469	736	-269	64
2015	532	751	-219	71
2016	573	763	-190	75
2017	609	778	-169	78
2018	627	791	-164	79
2019	661	806	-145	82
2020	673	824	-187	82
2021	703	836	-133	84

Table 16 shows the costs of rice production and distribution under organic and conventional cultivation. By comparing the various costs of organic and conventional rice cultivation, it can be found that the machine harvesting cost of organic rice is lower than that of conventional rice. At the same time, because organic farming uses more environmentally friendly and natural planting methods, it reduces the use of chemical pesticides and chemical fertilizers and saves 700 yuan. However, the cost of organic fertilizers and biopesticides is higher than that of conventional fertilizers and pesticides by the corresponding 250 yuan and 15 yuan. In addition, the price of organic seeds has increased by 50 yuan compared with conventional seeds. The cost of organic certification and product packaging and sales is also 200 and 500 yuan more than conventional rice, respectively. Overall, the total cost of organic rice is $454 more than conventional rice, but it can also be seen that organic rice has less impact on the environment and human health by reducing the use of chemical pesticides and fertilizers.

**Table 16:** Cost of rice production for sale under organic and conventional cultivation.

Item	Organic (RMB/mu)	Conventional (RMB/mu)	Organic vs Conventional (RMB/mu)
Machine Transplanting	90	90	0
Machine Harvesting	80	110	-30
Organic Fertilizer	250	0	250
Chemical Fertilizer	0	400	-400
Biological Pesticide	15	0	15
Chemical Pesticide	0	300	-300
Seeds	350	300	50
Manual Labor	600	600(0)	0(600)
Land Rent	700	700(0)	0(700)
Organic Certification	200	0	200
Rice Storage and Transportation	400	400	0
Product Packaging and Sales	700	0	500
Total Cost	3385	2900(1600)	484(1785)

Note: The data in brackets do not include the rent of farmers’ own land and the wages of farmers’ own labor

From an economic point of view, the cost of organic rice is usually higher than that of conventional rice. However, the market for organic products has been growing rapidly over the past few years. This has been largely driven by the growing consumer concern for health, environment and sustainability. Moreover, organic rice needs to follow strict production standards and be certified by third-party organizations. These standards and certifications ensure the quality and traceability of organic products and enable consumers to trust the authenticity and quality of organic rice. According to a survey, the price of organic rice in the market is about 20 yuan or even more, compared to about 2 yuan for conventional rice. This helps to improve economic efficiency. In addition, organic rice cultivation can reduce environmental pollution and land degradation, and in the long run, it can also achieve sustainable agricultural development. Therefore, despite the higher cost of organic rice, it can bring more economic benefits in the long run [88, 89].

## 6 Conclusions and policy recommendations

### 6.1 Conclusion

Organic rice farming reduces carbon emissions and also improves soil quality, maintains water quality, and promotes biodiversity, among many other ecological benefits. Therefore, the adoption of organic agriculture to grow rice can effectively reduce the negative impact of agriculture on climate change and help achieve the two-carbon goal. Using provincial panel data from 2011 to 2021, this paper empirically analyzed the impact of organic rice cultivation on eco-efficiency and its impact on achieving the dual-carbon target. The main conclusions of the study are as follows: (i) Organic rice cultivation shows significant economic benefits, with an annual GDP return of 40%, while the environmental benefits are good, and the carbon emission per 10,000 GDP is only 6.23 tons. The industry is in the stage of incremental economies of scale, and the high growth rate of capital investment shows a high return on assets. (ii) From 2011 to 2021, the eco-efficiency of organic rice showed an upward trend, especially reaching its highest point in 2019, indicating that organic rice cultivation has a positive feedback effect, that is, the better the planting condition, the stronger the boost to the entire industry. (iii) Urbanization and changes in planting area: The increase of urbanization rate increases the planting area of organic rice, while reducing the planting area of traditional rice, reflecting the role of urbanization in promoting agricultural transformation and high-quality development. (iv) Promoting the dual-carbon target: Organic rice farming can contribute positively to achieving the dual-carbon target, especially in small and medium-sized agricultural production, which can optimize environmental and economic benefits.

The main contributions of this study include: (i) improving the evaluation system of agricultural eco-efficiency, and by analyzing the eco-efficiency of organic rice, the research has enriched the theory of agricultural eco-efficiency and supported the in-depth understanding of sustainable agricultural development. (ii) Promote the achievement of dual-carbon targets, demonstrate the effectiveness of organic rice production in reducing greenhouse gas emissions and enhancing carbon sinks, and provide practical guidance for the green transformation of agriculture. (iii) The study indicates that the organic rice growing industry is in a growth phase and has a high rate of capital input growth. This means that the organic rice growing industry has potential and development prospects, and further research and attention are needed. The findings of this study provide policymakers, agricultural producers and researchers with important information about the organic rice growing industry, which can contribute to the sustainable development of agricultural industry and the optimization of capital allocation. (iv) The study examines the relationship between organic rice cultivation and urbanization and finds that increased urbanization will significantly promote organic rice cultivation, but will also significantly reduce conventional rice cultivation area. This finding reveals the impact of urbanization on agricultural industry structure and agricultural product selection. This has important implications for agricultural policy makers and agricultural market participants, providing direction on how agricultural transformation and agricultural supply can adapt to the changing demands brought about by urbanization.

### 6.2 Policy recommendations

Combined with the previous analysis, this paper proposes policies in three aspects: “promotion of organic rice cultivation”, “substitution of organic rice for conventional rice”, and “promotion of organic agriculture and dual carbon goals”. This paper proposes policy recommendations in three areas.

Promoting organic rice cultivation: The Government should implement diversified support strategies to ensure long-term sustainability. In the early stage of industrial development, the government can support the organic rice industry by directly giving financial subsidies to organic rice growers. This includes subsidizing the cost of organic certification, promoting organic farming techniques and setting up specialized loan programs. Government investment funds should also be used for organic rice market development and promotion activities, including publicity, brand building, exhibition exchanges, etc., in order to improve the visibility and market share of organic rice. Governments should also consider encouraging private sector involvement to support the development of organic rice cultivation through public-private partnership models. Effectively promote organic rice cultivation while ensuring its long-term economic and environmental sustainability through the implementation of a comprehensive and dynamically adjusted policy framework.

Substitution of organic rice for traditional rice: Given the dual advantages of organic rice cultivation in terms of economic and environmental effects and the urbanization process driving organic rice cultivation, the government can take measures such as financial subsidies, price subsidies, and yield guarantees to encourage farmers to gradually shift from traditional rice cultivation to organic rice cultivation [89–91]. In addition, the government can encourage farmers to organize local cooperatives and professional cooperatives to jointly promote organic rice cultivation, create large-scale production and improve market competitiveness.

Promoting organic farming and dual carbon goals: By combining the advantages of organic rice in economic and environmental dimensions, other types of organic farming may also have positive economic and environmental benefits [23, 92, 93]. The government can increase financial support for organic agriculture, support the development and promotion of organic farming technologies, and encourage farmers to adopt organic farming techniques. In addition, the government can establish a corresponding policy framework and standard system to regulate and certify organic farming production to ensure its quality and credibility [94]. At the same time, the government can adopt corresponding incentives to encourage enterprises and institutions to invest in the organic agriculture industry chain to form a virtuous cycle of the industrial ecosystem.

## Supporting information

S1 File (ZIP)
