# Supplementary material for: Chinese organic rice transition spatial econometrics empirical analysis
Source: PLoS One. 2024 Apr 11;19(4):e0297784. doi: 10.1371/journal.pone.0297784 (PMC11008895; doi:10.1371/journal.pone.0297784)
Supplement: S1 File — (ZIP) [file pone.0297784.s001.zip › Supporting Information/Appendix.docx]

**Appendix**

**Appendix 1** Qualitative Description of Variables.

| Variable symbol | Meaning | Measurement indicator and description | Unit |
| --- | --- | --- | --- |
| OS | Ownership structure | Non-state-owned fixed asset investment as a proportion of total fixed asset investment | % |
| ES | Energy consumption structure | Coal consumption as a proportion of total energy consumption | % |
| UR | Population structure | Urban population as a proportion of total population | % |
| TI | Green technology innovation capacity | Number of green patents | Items |
| EE | Energy efficiency | Energy consumption per unit of GDP | 100 million yuan/10,000 tons |
| FD | Fiscal decentralization | The financial calculation formula for an important indicator reflecting the size of local government fiscal autonomy: FD = per capita fiscal expenditure at the provincial level/ per capita fiscal expenditure at the central level | % |
| ER | Environmental regulatory level | The "governance input" indicator for economic regulation represents the investment amount in industrial pollution control | 10,000 yuan |
| TEC | Technological level | The number of patent applications and authorizations in each province and city are used as indicators | Individuals |
| FDI | Investment openness | The proportion of actual foreign direct investment utilized in GDP is used as an indicator | % |
| TRADE | Trade openness | The proportion of urban population to total population is used as an indicator |  |
| UL | Urbanization level | The proportion of urban population to total population is used as an indicator | % |
